# Association of TMPRSS2-ERG gene fusion with clinical characteristics and outcomes: results from a population-based study of prostate cancer

**DOI:** 10.1186/1471-2407-8-230

**Published:** 2008-08-11

**Authors:** Liesel M FitzGerald, Ilir Agalliu, Karynn Johnson, Melinda A Miller, Erika M Kwon, Antonio Hurtado-Coll, Ladan Fazli, Ashish B Rajput, Martin E Gleave, Michael E Cox, Elaine A Ostrander, Janet L Stanford, David G Huntsman

**Affiliations:** 1 Division of Public Health Sciences, Fred Hutchinson Cancer Research Center, 1100 Fairview Ave N., Seattle, WA 98109, USA; 2 Department of Epidemiology and Population Health, Albert Einstein College of Medicine, Bronx, NY 10461, USA; 3 The Prostate Research Centre, Vancouver General Hospital, Vancouver, BC V6H-3Z6, Canada; 4 The Centre for Translational and Applied Genomics, British Columbia Cancer Agency, Vancouver, BC V5Z-4E6, Canada; 5 National Human Genome Institute, Cancer Genetics Branch, National Institutes of Health, Building 50, 50 South Drive, Bethesda, MD 20892, USA; 6 Department of Epidemiology, School of Public Health and Community Medicine, University of Washington, Seattle, Washington, USA

## Abstract

**Background:**

The presence of the TMPRSS2-ERG fusion gene in prostate tumors has recently been associated with an aggressive phenotype, as well as recurrence and death from prostate cancer. These associations suggest the hypothesis that the gene fusion may be used as a prognostic indicator for prostate cancer.

**Methods:**

In this study, fluorescent *in situ *hybridization (FISH) assays were used to assess TMPRSS2-ERG fusion status in a group of 214 prostate cancer cases from two population-based studies. The FISH assays were designed to detect both fusion type (deletion vs. translocation) and the number of fusion copies (single vs. multiple). Genotyping of four *ERG *and one *TMPRSS2 *SNPs using germline DNA was also performed in a sample of the cases (n = 127).

**Results:**

Of the 214 tumors scored for the TMPRSS2-ERG fusion, 64.5% were negative and 35.5% were positive for the fusion. Cases with the TMPRSS2-ERG fusion did not exhibit reduced prostate cancer survival (HR = 0.92, 95% CI = 0.22–3.93), nor was there a significant difference in cause-specific survival when stratifying by translocation or deletion (HR = 0.84, 95% CI = 0.23–3.12) or by the number of retained fusion copies (HR = 1.22, 95% CI = 0.45–3.34). However, evidence for reduced prostate cancer-specific survival was apparent in those cases whose tumor had multiple copies of the fusion. The variant T allele of the *TMPRSS2 *SNP, rs12329760, was positively associated with TMPRSS2-ERG fusion by translocation (p = 0.05) and with multiple copies of the gene fusion (p = 0.03).

**Conclusion:**

If replicated, the results presented here may provide insight into the mechanism by which the TMPRSS2-ERG gene fusion arises and also contribute to diagnostic evaluations for determining the subset of men who will go on to develop metastatic prostate cancer.

## Background

There is considerable interest in the relationship between the TMPRSS2-ERG gene fusion and prostate cancer risk. Two studies, published in 2005, identified *ERG *as the most over-expressed proto-oncogene in prostate cancer tumors [1] and demonstrated that this over-expression is often caused by a fusion of the promoter region of the *TMPRSS2 *gene to a variety of genes [2]. Tomlins and colleagues (2005) identified recurrent gene fusions of *TMPRSS2 *to two *ETS *transcription factors, *ERG *and *ETV1*, and found evidence to suggest that these fusions may occur in the majority of prostate cancer cases [2].

Several subsequent studies have suggested that the TMPRSS2-ERG fusion protein is not only present in late stage prostate cancer [2-8] but in benign prostatic hyperplasia (BPH) [9], high-grade prostate intra-epithelial neoplasia (HGPIN) [3,10] and even in non-malignant tissue adjacent to prostate cancer foci [9,10]. During the course of these investigations, it has been discovered that there are a large number of unique TMPRSS2-ERG fusion transcripts, with up to 19 identified to date [7,9,11]. Interestingly, the majority of these transcripts, including the most commonly found T1/E4 variant, encode either truncated or null fusion proteins [7,9,11]. While some of the diversity may be due to alternative splicing, it has become apparent that other recombination mechanisms may also contribute to the distinct fusion transcripts. Several studies using FISH have demonstrated that the TMPRSS2-ERG fusion can result from both translocations and interstitial deletions between *TMPRSS2 *and *ERG *[5,11-14], with deletion being suggested as a common mechanism for fusion formation [11,15,16]. In addition, it has been demonstrated that while individual tumor foci are homogeneous for fusion status, within a single case heterogeneity between tumor foci also exists [8,9,17].

Several studies have focused on elucidating the role of the TMPRSS2-ERG gene fusion in prostate cancer. The gene fusion has been found to be associated with moderate to poorly differentiated prostate tumors [18], disease recurrence [4], progression and prostate cancer-specific death [15,19], and conversely, longer progression-free survival [20,21]. When investigating the many TMPRSS2-ERG isoforms, Wang and colleagues (2006) found expression of isoforms in which the native *TMPRSS2 *or *ERG *ATG start codon is in-frame are associated with aggressive disease and poor outcomes compared to non-native internal ATGs [7]. In addition, when investigating the type of TMPRSS2-ERG fusion, formation through deletion, rather than translocation, was associated with risk factors for disease progression [5] and significantly worse cause-specific and overall survival [15]. The latter study also found that deletion accompanied by duplication of the TMPRSS2-ERG fusion exhibited extremely poor cause-specific survival, providing prognostic information additional to that provided by Gleason score and PSA levels [15]. In fact, multiple studies have found no association between TMPRSS2-ERG fusion and Gleason score [8,11,14,17,20,22,23] or pathologic stage [8,11,14,20,23]. Further insight was provided by Hermans and colleagues (2006), who suggest that TMPRSS2-ERG may play a key role in androgen-dependent prostate cancer. While both androgen-dependent and androgen-independent tumors contain the fusion gene, only the former show overexpression of ERG and the fusion transcripts [12].

The main objective of the current study was to determine whether the TMPRSS2-ERG gene fusion was associated with prostate cancer-specific mortality in tumors from 372 patients ascertained from a population-based cancer registry and with long-term surveillance after cancer diagnosis. The second objective of this study was to investigate whether germline single nucleotide polymorphisms (SNPs) present in the *ERG *and *TMPRSS2 *genes are associated with TMPRSS2-ERG fusion status.

## Methods

### Study Subjects and Tumor Tissue

Tumor tissue blocks were collected from either radical prostatectomy specimens (n = 355, 95.4%) or TURP (n = 17, 4.6%), and were used to create tissue microarrays (TMAs). Patients from King County, Washington, were diagnosed with histologically confirmed prostate cancer from January 1, 1993 through December 31, 1996, and were identified via the Seattle-Puget Sound SEER Cancer Registry. The SEER registry provided information on Gleason score, stage of cancer, diagnostic PSA level and primary therapy. Vital status and underlying cause of death were also ascertained through the SEER cancer registry and collection of death certificates; November 15, 2007 was the most recent update of patient outcomes. The patients originate from two different studies. The first group of 270 men, diagnosed between the ages of 40 and 64 years, is part of a larger population-based case-control study consisting of 753 cases (subsequently referred to as Study I). The remaining 102 men, diagnosed between the ages of 60 and 88 years, are a subset of 372 patients who participated in a quality of life study (subsequently referred to as Study II). All patients signed informed consent for participation and the studies were approved by the Fred Hutchinson Cancer Research Center Institutional Review Board. Both of these studies are described in detail elsewhere [24-26].

### Tissue Microarrays and FISH Assay

**Figure 1:**
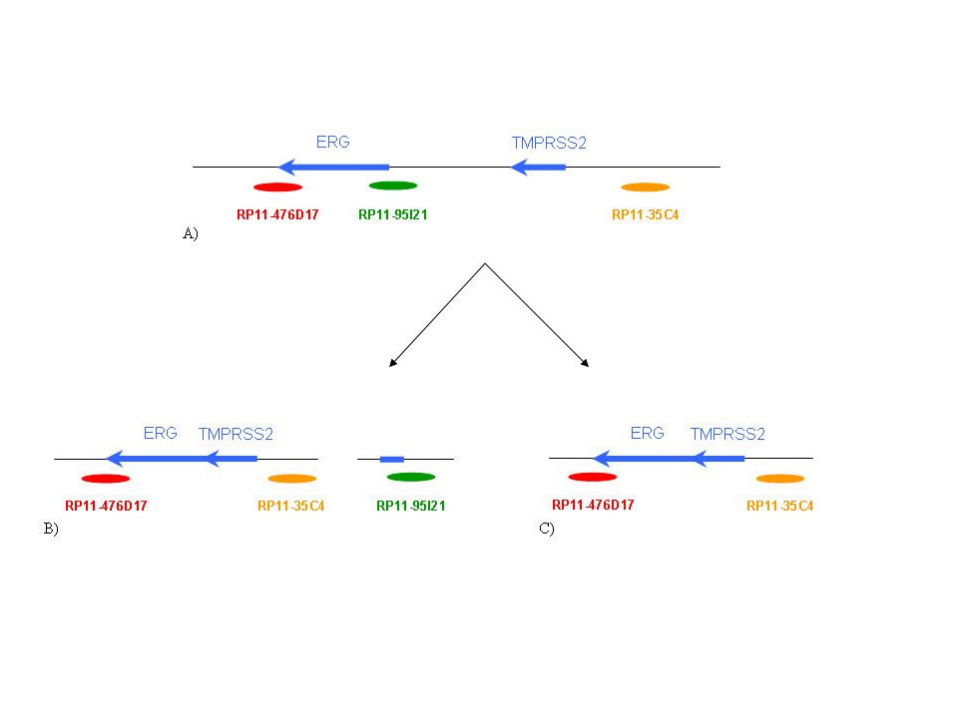
**Relative locations of BAC probes on chr21q22 for FISH assays**. A) Normal chromosome. B) TMPRSS2-ERG fusion by translocation. C) TMPRSS2-ERG fusion by deletion.

**Figure 2:**
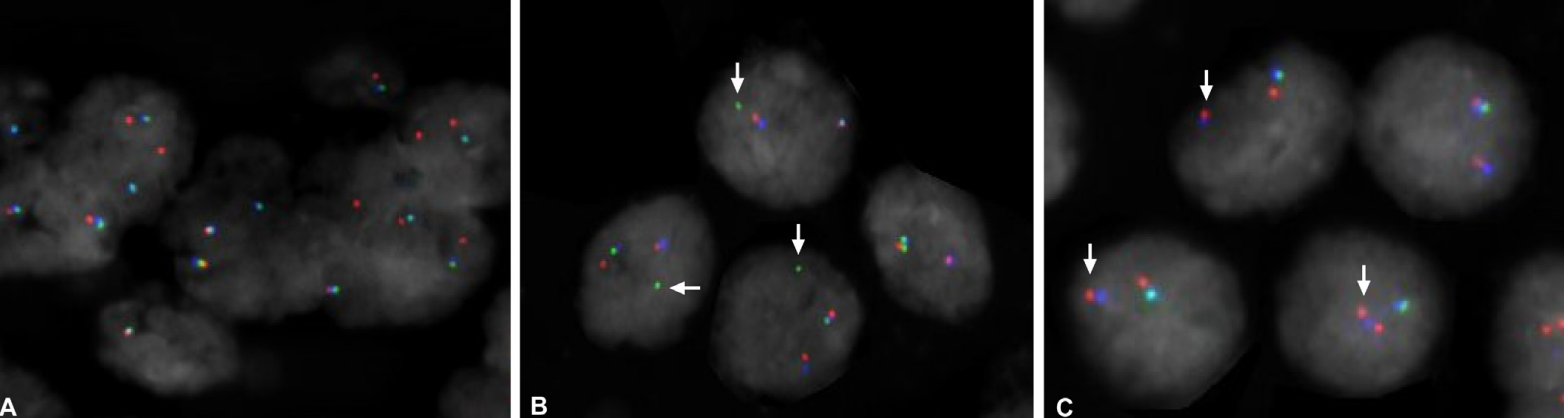
**FISH detection of *TMPRSS2 *and *ERG *gene status**. A) No TMPRSS2-ERG fusion. B) Positive for the TMPRSS2-ERG fusion with translocation – arrows indicate the separate green 5' *ERG *signal. C) Positive for the TMPRSS2-ERG fusion with deletion – arrows indicate the combined blue/red *TMPRSS2 *and 3' *ERG *signal and the absence of the green 5' *ERG *signal. FISH signals in these pseudo-colored images are red (telomeric to *TMPRSS2*; RP11-35C4), green (5' *ERG*; RP11-95I21) and blue (3' *ERG*; RP11-476D17). Note that in non-rearranged chromosomes the proximity of the green and blue signals can result in an aqua-colored signal.

A total of 372 paraffin embedded tumor blocks was used to build the TMAs. Of these specimens, eight were selected as blind duplicates for quality control purposes resulting in 380 cores being represented on the TMAs. H&E stained slides were reviewed for each case and areas containing tumor were marked on both the slides and corresponding paraffin blocks for TMA construction. Three cores per case, taken from a single tumor focus, were represented on the TMAs. The TMAs were constructed using a manual arrayer (Beecher Instruments) with tissue core diameters of 0.6 mm. Each section was baked overnight at 60°C, then deparaffinized in xylene and rinsed with 100% ethanol. Sections were pretreated as described previously [27]. FISH analysis was performed using the following BACs (BACPAC Resources Centre, Children's Hospital Oakland Research Institute, Oakland, CA): RP11-95I21 (5' ERG), RP11-476D17 (3' ERG) and RP11-35C4 (telomeric to TMPRSS2; Figure 1). BACs RP11-95I21 and RP11-35C4 were directly labeled by nick translation with Spectrum Green and Spectrum Orange respectively (Vysis, Downer's Grove, IL). BAC RP11-476D17 was indirectly labeled using a modified protocol with Streptavidin-Cy5 (MetaSystems, Belmont, MA) using the BioPrime DNA labeling system (Invitrogen). Probe labeling and FISH were performed using Vysis or MetaSystems reagents according to manufacturers' protocols. FISH signals were visualized on a Zeiss Axioplan epifluorescent microscope and captured using MetaSystems ISIS FISH imaging software (MetaSystems, Belmont, MA). Evaluation of the FISH results from each case was independently performed by 2 operators (KJ, MM). A total of 50 epithelial nuclei per case was evaluated across the three cores and to be classed positive for the TMPRSS2-ERG fusion, evidence needed to be present in at least 20% of the cells. Each individual was scored as follows (Figure 1 and 2): normal (three combined signals indicating no rearranged *TMPRSS2 *or *ERG *loci); positive for the fusion with translocation (combined red/blue signals with separate green 5' *ERG *signal); positive for the fusion with deletion (combined red/blue signals with the absence of the green 5' *ERG *signal); or not scored (absence of a FISH signal, low cellularity or core(s) dislodged from array). It was also noted whether multiple copies of the particular TMPRSS2-ERG fusion were present in a single nucleus.

### ERG and TMPRSS2 SNP Genotyping

Four SNPs within the *ERG *gene and one SNP within the *TMPRSS2 *gene were selected for genotyping using germline DNA isolated from peripheral blood samples using standard methods. The *ERG *SNPs were chosen based on results obtained from the CGEMS study (p ≤ 0.05) [28] and included rs1571704, rs1892570, rs2068967 and rs2836370. The *TMPRSS2 *SNP, rs12329760 (Met160Val), was chosen as it was previously found to be associated with prostate cancer in men with a first-degree family history of the disease [29]. Genotyping data were only available for a subgroup (N = 126) of Study I participants.

The Applied Biosystems (ABI) SNPlex™ Genotyping System was used for genotyping and proprietary GeneMapper^® ^software was used for allele calling [30]. Discrimination of the specific SNP allele was carried out on the ABI 3730*xl *DNA Analyzer and is based on the presence of a unique sequence assigned to the original allele-specific oligonucleotide. In 140 blind duplicate samples distributed across all genotyping batches, there was 100% agreement between the blinded samples for each of the five SNPs.

### Statistical Analysis

Demographic and clinical characteristics were compared between prostate cancer cases whose tumor could not be scored and those who were positive or negative for the TMPRSS2-ERG fusion. The primary endpoint for the survival analyses was time to death from prostate cancer. Survival time, i.e., time elapsed from diagnosis until death, was the time-dependent variable used. In each case, a death certificate was obtained to confirm the event. Living cases were censored as of November 15^th^, 2007. The association between prostate cancer specific-survival and TMPRSS2-ERG fusion status was evaluated using Kaplan-Meier estimator functions and Cox's proportional hazard models [31] to estimate hazard ratios (HR) and 95% confidence intervals (CI). First, survival analysis models were examined adjusting only for age at diagnosis, and then models were adjusted for Gleason score. Associations between genotyping data and TMPRSS2-ERG fusion status were examined using Fisher's exact test. All reported p-values were two-sided and SAS V9.1 was used for statistical analyses.

## Results

### Demographic and Clinical Results

Demographic and clinical characteristics of patients are provided in Table 1. Of the 372 unique prostate tumors, 214 (57.4%) were scored for the TMPRSS2-ERG fusion, one tumor (0.3%) was equivocal and 157 (42.4%) tumors were not scored due to technical issues (core drop-off or failed hybridization). There were no significant differences in age at diagnosis or clinical characteristics between those cases not scored and those scored for the TMPRSS2-ERG fusion. Of the 8 replicates present on the TMAs, equivalent results were obtained in the 4 samples with results from both replicates. After removing one of each replicate pair, 138 (64.5%) tumors scored negative and 76 (35.5%) tumors scored positive for the TMPRSS2-ERG fusion (Table 1). Of the tumors scored positive for the fusion transcript, 38 (50%) were fusion by translocation and 38 (50%) were fusion by deletion of the intervening chromosomal region (Table 1). There were no differences in the distributions of demographic or clinical characteristics between cases positive for the TMPRSS2-ERG fusion and those negative for the fusion. However, a higher proportion of prostate cancer-specific deaths was observed in cases whose tumor was positive for the TMPRSS2-ERG fusion in comparison to those without, 7.9% vs. 4.4% respectively (p = 0.35).

**Table 1:** Demographic and clinical characteristics of prostate cancer patients by tumor TMPRSS2-ERG gene fusion status.

	**TMPRSS2-ERG Gene Fusion Status**		
**Characteristics**	**Not Scored**^1^	**Positive**	**Negative**
	n = 158	n = 76	n = 138
Age at diagnosis			
Mean (SD)	61.2 (7.4)	59.3 (6.3)	59.9 (7.2)
Range	40 – 86	45 – 88	45 – 86
Gleason score (%)			
2 - 7 (3+4)	138 (87.3)	63 (82.9)	115 (83.3)
7 (4+3) - 10	17 (10.8)	12 (15.8)	19 (13.8)
Missing	3 (1.9)	1 (1.3)	4 (2.9)
Stage (%)			
Local	121 (76.6)	57 (75)	106 (76.8)
Regional	37 (23.4)	18 (23.7)	29 (21.0)
Distant	0 (0)	1 (1.3)	3 (2.2)
PSA value at diagnosis (ng/ml) (%)			
0 – 9.9	92 (58.2)	52 (68.4)	91 (65.9)
≥ 10.0	41 (26.0)	16 (21.1)	29 (21.0)
Missing	25 (15.8)	8 (10.5)	18 (13.1)
Vital status (%)			
Alive	129 (81.7)	65 (85.5)	119 (86.2)
Deceased			
Prostate cancer-specific death	4 (2.5)	6 (7.9)	6 (4.4)
Other cause of death	25 (15.8)	5 (6.6)	12 (8.7)
Missing	0 (0)	0 (0)	1 (0.7)
Fusion Type			
Translocation	-	38 (50)	-
Deletion	-	38 (50)	-
Fusion Number			
Single	-	65 (85.5)	-
Multiple	-	11 (14.5)	-
Survival Time (years) (SD)	11.5 (2.6)	12.0 (2.6)	11.4 (3.1)

^1^Includes one equivocal case.

### Survival Analysis

**Figure 3:**
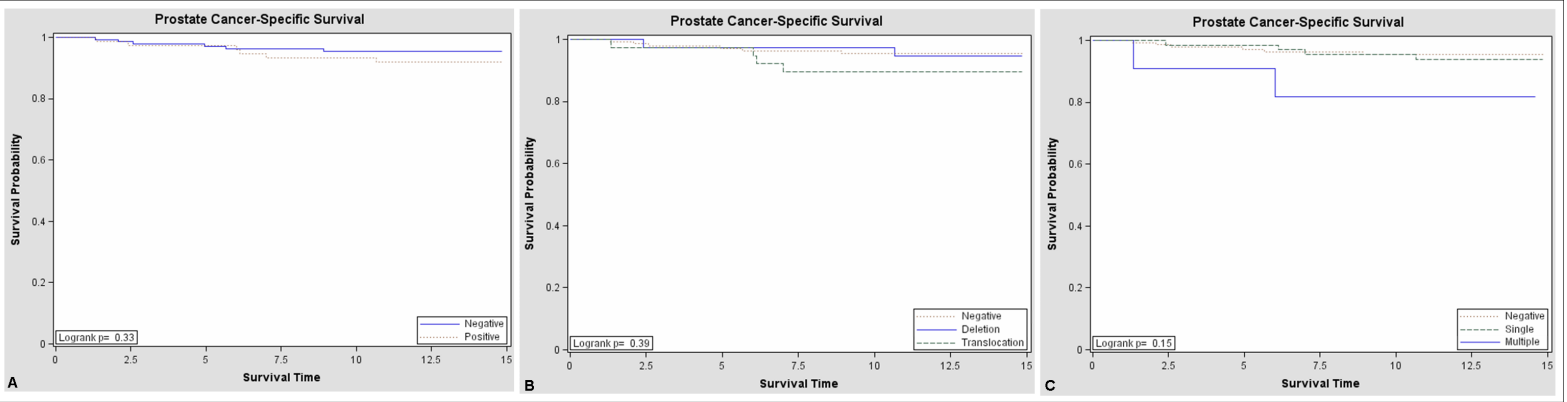
**Kaplan-Meier survival estimates**. A) Patients with and without TMPRSS2-ERG gene fusion. B) Patients with no TMPRSS2-ERG fusion, patients with fusion by translocation and patients with fusion by deletion. C) Patients with no TMPRSS2-ERG fusion, patients with a single fusion and patients with multiple fusions.

Prostate cancer-specific survival was evaluated for the 214 cases scored for the TMPRSS2-ERG fusion and these results are summarized in Kaplan-Meier plots (Figure 3). The mean survival time for all cases after diagnosis was 11.6 years (range 1 to 14 years). The mean survival time did not differ significantly between cases positive or negative for the TMPRSS2-ERG fusion, between cases with different fusion types (translocation vs. deletion) or between cases with different fusion copy numbers (single vs. multiple; Table 2). Multivariate Cox analysis, adjusted for age, demonstrated that a reduced, but not significant, cause-specific survival was observed in patients with fusion positive tumors, when compared to patients whose samples retain normal *TMPRSS2 *and *ERG *FISH patterns (HR = 2.4, 95% CI = 0.74–7.57). However, after adjusting for Gleason score the hazard ratio was substantially attenuated (HR = 1.2, 95% CI = 0.34–4.02; Table 2). Similarly, no significant differences in cause-specific survival were observed when stratifying the fusion samples by translocation or deletion (HR = 1.18, 95% CI = 0.52–2.71; Table 2), or by the number of retained copies (HR = 1.46, 95% CI = 0.54–3.88; Table 2). However, even after adjusting for age and clinicopathological factors, there was some evidence that samples with multiple copies of the fusion conferred worse survival than those with no fusion or single copies of the gene fusion.

**Table 2:** Patient mean survival time and Cox regression analysis of TMPRSS2-ERG fusion status

**Fusion Status**	**Survival Time (SD)**^1^	**HR (95% CI)**^2^	**p-value**	**HR (95% CI)**^3^	**p-value**
Negative	11.4 (3.1)				
Positive	12.0 (2.6)	2.4 (0.7 – 7.6)	0.3	1.2 (0.3 – 4.0)	0.8
					
Negative	11.4 (3.1)				
Translocation	12.0 (2.9)				
Deletion	12.0 (2.2)	1.5 (0.7 – 2.9)	0.3	1.2 (0.5 – 2.7)	0.5
					
Negative	11.4 (3.1)				
Single	12.1 (2.3)				
Multiple	11.5 (4.1)	1.9 (0.9 – 4.1)	0.09	1.5 (0.6 – 3.9)	0.9

^1^Time elapsed from diagnosis until death; ^2^Age adjusted; ^3^Age & Gleason score adjusted.

### ERG and TMPRSS2 Genotyping Results

Examination of the associations between *ERG *and *TMPRSS2 *genotypes and the presence of the fusion gene, fusion type and fusion copy number are presented in Table 3. Polymorphisms present in the *ERG *gene were not related to the presence of the gene fusion, fusion type (translocation vs. deletion) or fusion copy number (single vs. multiple; Table 3). Presence of the variant allele of the *TMPRSS2 *SNP, rs12329760, was not associated with the presence of the TMPRSS2-ERG fusion (Fisher's Exact test; p = 0.13; Table 3), but men with a variant T allele were more likely to have fusion by translocation (χ^2 ^test; p = 0.05; Table 3) and to have multiple copies of the gene fusion (χ^2 ^test; p = 0.03; Table 3).

**Table 3:** Association between ERG and TMPRSS2 SNP genotypes and TMPRSS2-ERG fusion status

			**Fusion Status**			
				
**Gene**	**SNP**	**Genotype**	**Negative**	**Positive**		**P value**
ERG	rs1571704	GG	66 (84.6)	43 (89.6)		
		GT/TT	12 (15.4)	5 (10.4)		0.59
	rs1892570	CC	64 (81.0)	44 (91.7)		
		CT/TT	15 (19.0)	4 (8.3)		0.13
	rs2068967	GG	24 (30.4)	15 (31.3)		
		GA/AA	55 (69.6)	33 (68.7)		0.10
	rs2836370	TT	18 (22.5)	12 (25.5)		
		TC/CC	62 (77.5)	35 (74.5)		0.83
TMPRSS2	rs12329760	CC	53 (68.0)	26 (53.1)		
		CT/TT	25 (32.0)	23 (46.9)		0.13
						
			**Negative**	**Translocation**	**Deletion**	
				
ERG	rs1571704	GG	66 (84.6)	18 (85.7)	25 (92.6)	
		GT/TT	12 (15.4)	3 (14.3)	2 (7.4)	0.66
	rs1892570	CC	64 (81.0)	19 (90.5)	25 (92.6)	
		CT/TT	15 (19.0)	2 (9.5)	2 (7.4)	0.33
	rs2068967	GG	24 (30.4)	9 (42.8)	6 (22.2)	
		GA/AA	55 (69.6)	12 (57.2)	21 (77.8)	0.31
	rs2836370	TT	18 (22.5)	5 (23.8)	7 (26.9)	
		TC/CC	62 (77.5)	16 (76.2)	19 (73.1)	0.91
TMPRSS2	rs12329760	CC	53 (68.0)	8 (38.1)	18 (64.3)	
		CT/TT	25 (32.0)	13 (61.9)	10 (35.7)	0.05
						
			**Negative**	**Single**	**Multiple**	
				
ERG	rs1571704	GG	66 (84.6)	40 (93.0)	3 (60.0)	
		GT/TT	12 (15.4)	3 (7.0)	2 (40.0)	0.09
	rs1892570	CC	64 (81.0)	40 (93.0)	4 (80.0)	
		CT/TT	15 (19.0)	3 (7.0)	1 (20.0)	0.15
	rs2068967	GG	24 (30.4)	12 (27.9)	3 (60.0)	
		GA/AA	55 (69.6)	31 (72.1)	2 (40.0)	0.36
	rs2836370	TT	18 (22.5)	11 (26.2)	1 (20.0)	
		TC/CC	62 (77.5)	31 (73.8)	4 (80.0)	0.86
TMPRSS2	rs12329760	CC	53 (68.0)	25 (58.1)	1 (16.7)	
		CT/TT	25 (32.0)	18 (41.9)	5 (83.3)	0.03

## Discussion

In the 214 prostate cancer patients scored for fusion status, we found that the presence of the TMPRSS2-ERG fusion was not associated with prostate cancer-specific mortality. Similarly, no statistically significant association was found between prostate cancer-specific mortality and fusion type (translocation vs. deletion) or number (single vs. multiple). However, there was a suggestion of higher prostate cancer-specific mortality in those patients with multiple fusion products. In addition, we found the rs12329760 SNP in *TMPRSS2 *to be significantly associated with fusion by translocation and with multiple copies of the fusion protein.

Currently, only two previous studies have investigated the relationship between fusion status and prostate cancer-specific mortality [15,19]. Consistent with the results presented here, Demichelis and colleagues (2007) observed no significant association between TMPRSS2-ERG gene fusion and disease-specific mortality when results were adjusted for Gleason score and age (p = 0.2) [19]. By comparison, Attard and colleagues (2007) did not present results for overall fusion status however, they did show that fusions caused by deletion had significantly worse disease-specific mortality and this association was largely driven by tumors with two or more copies of the fusion product [15]. While we also observed suggestive evidence for higher prostate cancer-specific mortality in patients with multiple fusion products, we did not observe an association with fusion type (translocation or deletion). This could be due to a number of differences between our study and Attard and colleagues' [15]. Although the follow-up time of this study was greater than that of Attard *et al. *(median 12.3 years vs. 7.5 years), there were fewer prostate cancer-specific deaths during this time that could be attributed to the younger median age of diagnosis (60 years) and fewer cases with a Gleason score of > 7. As Gleason score is a strong, independent predictor of adverse outcomes and we have a relatively young group of patients, it may require a longer follow-up time to observe a potential association between prostate cancer-specific death and TMPRSS2-ERG fusion type (translocation/deletion) or number (single/multiple).

While not statistically significant, when unadjusted for Gleason score, we found that there was some evidence for higher prostate cancer-specific mortality in patients with fusions caused by translocation but not deletion. Attard and colleagues (2007) suggest that the sequence intervening the *TMPRSS2 *and *ERG *genes may contain tumor-suppressor genes which when lost, increase disease aggressiveness [15]. In addition, Birger and colleagues (2006) identified significantly down-regulated genes located in the area of the common deletion site, of which at least one, *HMGN1*, has been associated with tumor growth in mice and primary mouse embryonic fibroblast cell lines [32]. While deletion of this intervening region could explain the poorer outcome, it is not implausible that translocation could also disrupt the expression of these intervening genes in such a way to cause adverse outcomes. Clearly more work needs to be done, first to determine whether one particular fusion type is associated with poor outcomes and second, to determine whether intervening genes do play a role in the biological effect of the TMPRSS2-ERG fusion.

Few studies have addressed the issue of how the TMPRSS2-ERG fusion is initiated in prostate carcinogenesis. Using a bioinformatics approach, Liu and colleagues (2007) found Alu repeats in the *TMPRSS2 *and *ERG *breakpoint regions and that the distribution of these repeats correlates with the structure of the multiple TMPRSS2-ERG fusion transcripts identified to date [22]. This finding as well as the fact that genomic alterations associated with Alu repeats have been observed to be associated with various other cancers, led Liu and colleagues (2007) to suggest that these Alu elements may facilitate recombination that leads to the fusion of the *TMPRSS2 *and *ERG *genes in prostate cancer. To our knowledge, this is the first study to investigate whether common genetic polymorphisms present in the *TMPRSS2 *and *ERG *genes in germline DNA are associated with the presence and/or type of TMPRSS2-ERG fusion in prostate tumor tissue. No associations were detected between the *ERG *SNPs and gene fusion, however we did find an association between the *TMPRSS2 *SNP rs12329760 and particular forms of the TMPRSS2-ERG fusion. While this finding needs to be replicated, it is interesting to speculate on how this SNP could influence the formation of the fusion protein. The Met160Val amino acid is highly conserved across mammals (ancestral form is the C allele or Val amino acid) [33] suggesting that it may be less tolerant to substitutions. The SNP is present in an exonic splicing enhancer (ESE) srp40 site and the presence of the A allele is predicted to disrupt the ESE, potentially resulting in an increased chance of exon skipping or protein malformation [34]. It is also interesting to speculate on whether other gene fusions are contributing to prostate cancer development and progression. There are a great number of proteins that have been found to be over or under-expressed in prostate cancer and to be associated with various stages of tumor development. It is possible that like the fusions between *TMPRSS2 *and *ERG*, *ETV1*, *ETV4 *and *ETV5*, other functionally identical fusions are involved in changes in gene expression and prostate cancer development but are yet to be discovered [2,35,36].

If the association between the *TMPRSS2 *SNP and fusion type is replicated and in particular, if other SNPs associated with the acquisition of this gene fusion are identified, these data may present opportunities to augment or further current prostate cancer diagnostic abilities. As the gene fusion has been associated with early forms of prostate cancer, a germline genetic test could be developed to augment current screening procedures. In addition, clinicians are currently unable to distinguish men who will go on to develop aggressive metastatic prostate cancer from those whose disease will remain indolent. This may change with recent suggestions that particular TMPRSS2-ERG fusion types are a predictor of aggressive disease and prostate cancer-specific mortality [4,5,7,15,19]. Again a genetic test may be able to alert clinicians to those men who are more at risk for aggressive disease and therefore treatment strategies could be tailored accordingly.

There are both advantages and limitations to this study that must be taken into consideration when interpreting the results. Cases in this study were population-based unlike several previous TMPRSS2-ERG studies [4,5,18,19], there was a mean surveillance period of 11.6 years after diagnosis, and prostate cancer-specific death was confirmed by death certificate. However, due to an average 5-year relative survival rate for prostate cancer of 98%, there were few prostate cancer-specific deaths in this cohort and therefore limited power. In addition, due to the technical problems inherent to assaying TMAs using FISH [37], only 57% of the cases could be scored. As a consequence, while there was some evidence of an association between multiple fusions and cancer-specific survival, there were insufficient events to observe a statistically significant association. Lack of power is also a concern in the SNP analyses and overall replication of the study is a priority before any translational studies are initiated. One final concern is that only one tumor focus was investigated per case in this study. As noted in the Introduction, focal heterogeneity is typically observed so it is possible cases were scored as normal when they did in fact have fusion transcripts present at other foci. Key future studies need to address the issue of whether results from one focus are predictive of tumor behavior overall.

## Conclusion

In summary, while no statistically significant associations were observed, the data presented here show a suggestive trend toward greater prostate cancer-specific mortality in men whose tumors have multiple copies of TMPRSS2-ERG. In addition, the *TMPRSS2 *SNP, rs12329760, was associated with multiple copies of TMPRSS2-ERG and fusion by translocation. These findings, if confirmed, may provide insight into the mechanism by which the fusion occurs and have an impact on the method of elucidating indolent from more aggressive prostate cancers.

## Competing interests

The authors declare that they have no competing interests.

## Authors' contributions

All authors read and approved the final manuscript. LMF carried out the analysis, interpretation of the data and manuscript writing. IA provided substantial input into the analysis and manuscript revision. KJ & MAM conducted the FISH experiments. EAO & EMK conducted the genotyping experiments. AH-C, LF, ABR & MEG provided pathology expertise and constructed the TMAs. MEC participated in the design and coordination of the study. EAO provided substantial input into the manuscript revision. JLS collected the patient data and tumor samples, participated in the design and coordination of the study and provided substantial input into the manuscript revision. DGH conceived of the study, participated in its design and coordination, and provided substantial input into the manuscript revision.

## Pre-publication history

The pre-publication history for this paper can be accessed here:



## References

[B1] Petrovics G, Liu A, Shaheduzzaman S, Furusato B, Sun C, Chen Y, Nau M, Ravindranath L, Chen Y, Dobi A, Srikantan V, Sesterhenn IA, McLeod DG, Vahey M, Moul JW, Srivastava S (2005). Frequent overexpression of ETS-related gene-1 (ERG1) in prostate cancer transcriptome. Oncogene.

[B2] Tomlins SA, Rhodes DR, Perner S, Dhanasekaran SM, Mehra R, Sun XW, Varambally S, Cao X, Tchinda J, Kuefer R, Lee C, Montie JE, Shah RB, Pienta KJ, Rubin MA, Chinnaiyan AM (2005). Recurrent fusion of TMPRSS2 and ETS transcription factor genes in prostate cancer. Science.

[B3] Cerveira N, Ribeiro FR, Peixoto A, Costa V, Henrique R, Jeronimo C, Teixeira MR (2006). TMPRSS2-ERG gene fusion causing ERG overexpression precedes chromosome copy number changes in prostate carcinomas and paired HGPIN lesions. Neoplasia.

[B4] Nam RK, Sugar L, Yang W, Srivastava S, Klotz LH, Yang LY, Stanimirovic A, Encioiu E, Neill M, Loblaw DA, Trachtenberg J, Narod SA, Seth A (2007). Expression of the TMPRSS2:ERG fusion gene predicts cancer recurrence after surgery for localised prostate cancer. Br J Cancer.

[B5] Perner S, Demichelis F, Beroukhim R, Schmidt FH, Mosquera JM, Setlur S, Tchinda J, Tomlins SA, Hofer MD, Pienta KG, Kuefer R, Vessella R, Sun XW, Meyerson M, Lee C, Sellers WR, Chinnaiyan AM, Rubin MA (2006). TMPRSS2:ERG fusion-associated deletions provide insight into the heterogeneity of prostate cancer. Cancer Res.

[B6] Soller MJ, Isaksson M, Elfving P, Soller W, Lundgren R, Panagopoulos I (2006). Confirmation of the high frequency of the TMPRSS2/ERG fusion gene in prostate cancer. Genes Chromosomes Cancer.

[B7] Wang J, Cai Y, Ren C, Ittmann M (2006). Expression of variant TMPRSS2/ERG fusion messenger RNAs is associated with aggressive prostate cancer. Cancer Res.

[B8] Mehra R, Han B, Tomlins SA, Wang L, Menon A, Wasco MJ, Shen R, Montie JE, Chinnaiyan AM, Shah RB (2007). Heterogeneity of TMPRSS2 gene rearrangements in multifocal prostate adenocarcinoma: molecular evidence for an independent group of diseases. Cancer Res.

[B9] Clark J, Merson S, Jhavar S, Flohr P, Edwards S, Foster CS, Eeles R, Martin FL, Phillips DH, Crundwell M, Christmas T, Thompson A, Fisher C, Kovacs G, Cooper CS (2007). Diversity of TMPRSS2-ERG fusion transcripts in the human prostate. Oncogene.

[B10] Furusato B, Gao CL, Ravindranath L, Chen Y, Cullen J, McLeod DG, Dobi A, Srivastava S, Petrovics G, Sesterhenn IA (2008). Mapping of TMPRSS2-ERG fusions in the context of multi-focal prostate cancer. Mod Pathol.

[B11] Tu JJ, Rohan S, Kao J, Kitabayashi N, Mathew S, Chen YT (2007). Gene fusions between TMPRSS2 and ETS family genes in prostate cancer: frequency and transcript variant analysis by RT-PCR and FISH on paraffin-embedded tissues. Mod Pathol.

[B12] Hermans KG, van Marion R, van Dekken H, Jenster G, van Weerden WM, Trapman J (2006). TMPRSS2:ERG fusion by translocation or interstitial deletion is highly relevant in androgen-dependent prostate cancer, but is bypassed in late-stage androgen receptor-negative prostate cancer. Cancer Res.

[B13] Iljin K, Wolf M, Edgren H, Gupta S, Kilpinen S, Skotheim RI, Peltola M, Smit F, Verhaegh G, Schalken J, Nees M, Kallioniemi O (2006). TMPRSS2 fusions with oncogenic ETS factors in prostate cancer involve unbalanced genomic rearrangements and are associated with HDAC1 and epigenetic reprogramming. Cancer Res.

[B14] Yoshimoto M, Joshua AM, Chilton-Macneill S, Bayani J, Selvarajah S, Evans AJ, Zielenska M, Squire JA (2006). Three-color FISH analysis of TMPRSS2/ERG fusions in prostate cancer indicates that genomic microdeletion of chromosome 21 is associated with rearrangement. Neoplasia.

[B15] Attard G, Clark J, Ambroisine L, Fisher G, Kovacs G, Flohr P, Berney D, Foster CS, Fletcher A, Gerald WL, Moller H, Reuter V, De Bono JS, Scardino P, Cuzick J, Cooper CS (2008). Duplication of the fusion of TMPRSS2 to ERG sequences identifies fatal human prostate cancer. Oncogene.

[B16] Mehra R, Tomlins SA, Shen R, Nadeem O, Wang L, Wei JT, Pienta KJ, Ghosh D, Rubin MA, Chinnaiyan AM, Shah RB (2007). Comprehensive assessment of TMPRSS2 and ETS family gene aberrations in clinically localized prostate cancer. Mod Pathol.

[B17] Barry M, Perner S, Demichelis F, Rubin MA (2007). TMPRSS2-ERG fusion heterogeneity in multifocal prostate cancer: clinical and biologic implications. Urology.

[B18] Rajput AB, Miller MA, De Luca A, Boyd N, Leung S, Hurtado-Coll A, Fazli L, Jones EC, Palmer JB, Gleave ME, Cox ME, Huntsman DG (2007). Frequency of the TMPRSS2:ERG gene fusion is increased in moderate to poorly differentiated prostate cancers. J Clin Pathol.

[B19] Demichelis F, Fall K, Perner S, Andren O, Schmidt F, Setlur SR, Hoshida Y, Mosquera JM, Pawitan Y, Lee C, Adami HO, Mucci LA, Kantoff PW, Andersson SO, Chinnaiyan AM, Johansson JE, Rubin MA (2007). TMPRSS2:ERG gene fusion associated with lethal prostate cancer in a watchful waiting cohort. Oncogene.

[B20] Saramaki OR, Harjula AE, Martikainen PM, Vessella RL, Tammela TL, Visakorpi T (2008). TMPRSS2:ERG Fusion Identifies a Subgroup of Prostate Cancers with a Favorable Prognosis. Clin Cancer Res.

[B21] Winnes M, Lissbrant E, Damber JE, Stenman G (2007). Molecular genetic analyses of the TMPRSS2-ERG and TMPRSS2-ETV1 gene fusions in 50 cases of prostate cancer. Oncol Rep.

[B22] Liu W, Ewing CM, Chang BL, Li T, Sun J, Turner AR, Dimitrov L, Zhu Y, Sun J, Kim JW, Zheng SL, Isaacs WB, Xu J (2007). Multiple genomic alterations on 21q22 predict various TMPRSS2/ERG fusion transcripts in human prostate cancers. Genes Chromosomes Cancer.

[B23] Lapointe J, Kim YH, Miller MA, Li C, Kaygusuz G, van de Rijn M, Huntsman DG, Brooks JD, Pollack JR (2007). A variant TMPRSS2 isoform and ERG fusion product in prostate cancer with implications for molecular diagnosis. Mod Pathol.

[B24] Penson DF, Feng Z, Kuniyuki A, McClerran D, Albertsen PC, Deapen D, Gilliland F, Hoffman R, Stephenson RA, Potosky AL, Stanford JL (2003). General quality of life 2 years following treatment for prostate cancer: what influences outcomes? Results from the prostate cancer outcomes study. J Clin Oncol.

[B25] Potosky AL, Reeve BB, Clegg LX, Hoffman RM, Stephenson RA, Albertsen PC, Gilliland FD, Stanford JL (2002). Quality of life following localized prostate cancer treated initially with androgen deprivation therapy or no therapy. J Natl Cancer Inst.

[B26] Stanford JL, Wicklund KG, McKnight B, Daling JR, Brawer MK (1999). Vasectomy and risk of prostate cancer. Cancer Epidemiol Biomarkers Prev.

[B27] Makretsov N, He M, Hayes M, Chia S, Horsman DE, Sorensen PH, Huntsman DG (2004). A fluorescence *in situ* hybridization study of ETV6-NTRK3 fusion gene in secretory breast carcinoma. Genes Chromosomes Cancer.

[B28] National Cancer Institute - Cancer Genetic Markers of Susceptibility National Cancer Institute - Cancer Genetic Markers of Susceptibility. http://cgems.cancer.gov/.

[B29] Lubieniecka JM, Cheteri MK, Stanford JL, Ostrander EA (2004). Met160Val polymorphism in the TRMPSS2 gene and risk of prostate cancer in a population-based case-control study. Prostate.

[B30] Biosystems A Applied Biosystems. http://www.appliedbiosystems.com.

[B31] Cox DR (1972). Regression models and life tables (with discussion).. J Royal Stat Soc B.

[B32] Birger Y, Catez F, Furusawa T, Lim JH, Prymakowska-Bosak M, West KL, Postnikov YV, Haines DC, Bustin M (2005). Increased tumorigenicity and sensitivity to ionizing radiation upon loss of chromosomal protein HMGN1. Cancer Res.

[B33] Bioinformatics UCSCG UCSC Genome Bioinformatics. UCSC Genome Bioinformatics.

[B34] Cartegni L, Wang J, Zhu Z, Zhang MQ, Krainer AR (2003). ESEfinder: A web resource to identify exonic splicing enhancers. Nucleic Acids Res.

[B35] Helgeson BE, Tomlins SA, Shah N, Laxman B, Cao Q, Prensner JR, Cao X, Singla N, Montie JE, Varambally S, Mehra R, Chinnaiyan AM (2008). Characterization of TMPRSS2:ETV5 and SLC45A3:ETV5 gene fusions in prostate cancer. Cancer Res.

[B36] Tomlins SA, Mehra R, Rhodes DR, Smith LR, Roulston D, Helgeson BE, Cao X, Wei JT, Rubin MA, Shah RB, Chinnaiyan AM (2006). TMPRSS2:ETV4 gene fusions define a third molecular subtype of prostate cancer. Cancer Res.

[B37] Brown LA, Huntsman D (2007). Fluorescent *in situ* hybridization on tissue microarrays: challenges and solutions. J Mol Histol.

